# Reading for life-long health

**DOI:** 10.3389/fped.2024.1401739

**Published:** 2024-07-24

**Authors:** Kaiulani Shulman, Karen Baicker, Linda Mayes

**Affiliations:** ^1^ Child Study Center, School of Medicine, Yale University, New Haven, CT, United States; ^2^ Scholastic Corporation, New York, NY, United States

**Keywords:** reading, physical health, mental health, early childhood education, health outcomes, literacy

## Abstract

There is a strong, positive relationship between childhood literacy and physical and mental health outcomes in adulthood. Through primary care-based literacy interventions, pediatricians reach children and their families long before they enter traditional education venues. In so doing, pediatricians play a key role in children's school readiness and in turn health outcomes. The current state of childhood literacy in United States defines an increasingly urgent platform for the healthcare profession generally, and pediatricians specifically, to embrace. Through reviewing the existing literature on the impact of childhood literacy on physical, mental, and social-emotional health outcomes, we hope to highlight the need for increased collaboration between the education and medical fields to further promote the literacy interventions in pediatric healthcare settings.

## Introduction

*The benefits of reading books include a longer life in which to read them* (1).

There is a strong, positive relationship between childhood literacy and physical and mental health outcomes in adulthood. Through primary care-based literacy interventions, pediatricians reach children and their families long before they enter traditional education venues. In so doing, pediatricians play a key role in children's school readiness and in turn health outcomes.

Research shows that access to books results in higher neurocognitive function and better physical health in adulthood (2–7). Children from homes that encourage literacy are more likely to achieve future academic success, as those with more books at home achieve, on average, three years more schooling, regardless of their parents’ education, occupation, and economic status (8). Perhaps most powerfully, a longitudinal study found that those who read books live almost 23 months longer than non-readers. Adults who read for more than 3.5 hours a week were 20 percent less likely to die over the 12-year study follow-up than those who didn't read books. Simply put: “The benefits of reading books include a longer life in which to read them.” (1).

The current state of childhood literacy in United States defines an increasingly urgent platform for the healthcare profession generally, and pediatricians specifically, to embrace. The 2022 National Assessment of Educational Progress (NAEP) findings reveal an unsettling portrait of childhood literacy trends in the United States. With 37% of fourth graders performing below the NAEP Basic level in reading, the percentage of students in the United States who achieve baseline literacy standards has decreased by approximately 14% since 1992, with a three-point loss since 2019—the largest drop in scores since NAEP testing first began. This drop in test scores span student race, income levels, and school type and location, disproportionately affecting students in the bottom 10th percentile nationwide, who are more likely to be from low-income communities and communities of color (9).

Though sobering, this decline in reading scores is not unexpected. Educators and families have raised concerns about literacy skills since March 2020, when the COVID-19 pandemic led to approximately 130,000 school closings in the United States, impacting 57 million children (10). During pandemic school closures, 93% of students nationwide engaged in some form of distance learning (11). Researchers hypothesize that 67% of kindergarten literacy skills were lost during the pandemic (12).

Through reviewing the existing literature on the impact of childhood literacy on physical, mental, and social-emotional health outcomes, we hope to highlight the need for increased collaboration between the education and medical fields to further promote the literacy interventions in pediatric healthcare settings.

## Literacy and children’s health

While it is never too late to encourage and foster literacy skills, the data highlight the long-term impact of commitment to literacy from the earliest ages, even before formal K–12 schooling has begun. The powerful influence of books and literacy reveals itself across a variety of metrics—from academic success to social-emotional skills to physical and mental health.

Educators know that home literacy environments are critical throughout childhood; by age 18, the average child in the United States will spend only 13% of their waking time in school. Furthermore, only 40% of U.S. three- and four-year-olds are enrolled in school (13).

Data suggest that reading interventions are most effective when administered to preschool-age children (14). Children who are read to at least three times a week at home are more likely to recognize each letter of the alphabet, count to 20, write their names, and read or pretend to read when they enter kindergarten (15). Additionally, research has found that children's vocabulary when entering first grade predicts their reading comprehension level in the eleventh grade (16). These advances hold true even as students move through formal educational settings: “If a child does not learn to read well within the first few years of school, then the chances of poor academic performance increase significantly” over the course of their schooling (17). While there is no doubt that reading skills and a commitment to literacy positively impact students’ future academic success as well as their long-term economic and social growth, research suggests that reading makes children healthier—physically and mentally.

### Physical health

When discussing the relationship between literacy and physical health, it is important to distinguish between educational attainment and literacy rates. While there is a body of research showing the correlation between educational attainment and health, data specifically related to literacy are less common.

A 2006 study at two primary-care clinics associated with San Francisco General Hospital offers promising insight into the role of literacy (18). Adult patients with type 2 diabetes mellitus, who graduated high school, had statistically improved glycemic control compared to those who had not graduated, but literacy mediated the association between educational achievement and glycemic control. Patients who exhibited high levels of reading skills were more likely to have improved glycemic control, regardless of whether they graduated from high school. In distinguishing between education and literacy, this study highlights the specific importance of reading skills in health outcomes (18).

Lower literacy rates are associated with poor physical-health outcomes: when controlling for demographic and socioeconomic factors, individuals with lower literacy rates are less likely to receive regular preventative healthcare measures such as pap smears, mammograms, and influenza and pneumococcal vaccinations (5, 6, 19, 20). In older children and adolescents, lower literacy rates are associated with higher risks of violence, aggressive behaviors, substance use, and sexually transmitted infections (21–26) Adults with lower literacy rates are at increased risk for hospitalization compared to their peers with higher literacy rates, both among those not receiving Medicare (1.69 times more likely to be hospitalized) and those receiving Medicare (1.2 times more likely) (5–7).

### Mental health

Childhood literacy is also associated with mental health outcomes. Beyond academic success and better physical health, students who struggle with reading are more likely to have internalized mental health conditions, such as anxiety and depression. Research suggests that self-esteem influences the relationship between reading skills and mental health, as children who have difficulty reading and low self-esteem exhibit more externalizing behaviors than their peers (27). Additionally, in 2018, the National Literacy Trust found that positive attitudes toward reading and writing were associated with improved resilience, motivation, self-esteem, and confidence, indicators of mental health. 37.4% of individuals with low literacy-engagement levels experience low mental health, while only 11.8% experience good mental health. Of those with high literacy-engagement levels, 39.4% experience good mental health (28).

A growing body of research indicates a relationship between reading fiction and affective empathy (29–31). One study found that exposure to fiction text is positively correlated with social ability, as “comprehending characters in a narrative fiction appears to parallel the compression of peers in the actual world.” (30) Further investigations suggest a positive correlation between fiction exposure, performance on empathy assessments, and social support (31, 32). Research suggests that “understanding others’ mental states is a crucial skill that enables the complex social relationships that characterize human societies,” adding that “reading uniquely engages the psychological processes needed to gain access to characters’ subjective experiences,” thereby increasing the reader's understanding of the world (33).

## Educators and pediatricians in partnership

Despite the well-established body of research showing the link between literacy and long-term health, education and medicine remain largely isolated sectors (34). However, over the past thirty years, organizations such as Reach Out and Read have begun bringing together educators and healthcare workers to establish literacy in dialogue with healthcare.

Founded in 1989 at Boston Medical Center to address disparities in literacy rates among low-income children, Reach Out and Read's design features three parts:

1. Pediatric medical professionals provide early literacy guidance to families at regular well-child appointments.
2. Children from birth to age five are provided a developmentally appropriate book at each office visit, with the aim of building an at-home library for each child.
3. Medical professionals provide each family with a “prescription” for 10 minutes of daily reading (35).

The results of early Reach Out and Read programs are impressive: The first research study found an eight-fold increase in parents reporting reading aloud as a favorite family activity —a powerful finding, as research indicates that joint parent-child reading increases children's language skills, with quantity and quality of that reading determining children's interest in reading as they age (36, 37). Today, Reach Out and Read serve 4.2 million children nationwide. Parents engaged in Reach Out and Read programs are 2.5 times more likely to read with their infants, toddlers, and preschoolers and 2 times more likely to read with their children three or more times per week. Reach Out and Read families are 2.5 times more likely to enjoy reading together or to have books in the home, and children's language development is improved by three to six months (35).

However, Programs such as Reach Out and Read cannot integrate literacy and healthcare alone. With fewer than half (48%) of poor children ready for school at age five, and only 75% of children from moderate- or high-income households prepared for Kindergarten, additional comprehensive interventions are necessary to ensure all children have the literacy skills to thrive physically, emotionally, and academically (38).

## Reading and literacy in healthcare

The American Academy of Pediatrics (AAP) has addressed the importance of literacy, recommending that providers promote early literacy development beginning in infancy and discuss early childhood education options with families (39). The AAP advises that physicians discuss specific strategies with parents and caregivers, including encouraging reading aloud with young children, discussing shared-reading activities, and providing developmentally appropriate books at health supervision visits for all “high-risk” low-income young children. However, there are no existing guidelines for pediatricians to assess literacy or to consider literacy-rich environments as an indicator for children's physical or mental health (39).

Numerous studies have examined the potential for instituting literacy interventions in pediatric healthcare settings. In one study of families with children between 6 and 38 months of age, researchers used a child-centered literacy orientation survey to examine the impact of providing relevant information on literacy promotion and children's books during pediatric primary-care visits (40). Parents and caregivers were asked to name their families’ and their children's favorite activities, and how many nights each week they shared books with their child before bedtime. Upon answering, they were assigned a score and placed in a research group. At the conclusion of the study, the results were stunning: families receiving the treatment intervention had five times the literacy orientation scores of the control group, and children 18 months or older whose parents participated in the intervention had higher language scores than their peers (40).

Furthermore, a study of low-income Hispanic parents of infants who received age-appropriate children's books from their pediatric healthcare providers showed similar results. With a 96% follow-up rate after 10 months, parents in the treatment intervention were 10 times as likely to read to their child three or more days per week, and to include reading among their three favorite parent-child activities (41). These studies demonstrate that delivering literacy interventions to children and their families in the pediatric healthcare setting has a positive impact on literacy.

## Literacy as a public health crisis

There is no denying that the United States’ literacy crisis poses significant health risks to our nation. According to the National Center for Educational Statistics (NCES), 21% of adults in the United States (about 43 million) fall into the illiterate/functionally illiterate category. Nearly two-thirds of fourth graders read below grade level, and the same number graduate from high school still reading below grade level (42). Furthermore, The American Journal of Public Health reports that the inability to read and understand health information accounts for $232 billion spent in healthcare costs each year, with studies linking low literacy to problems with use of preventive services, delayed diagnosis, adherence to medical instructions, amongst others (43). Therefore, we must acknowledge the “central role of the pediatrician in school readiness” and expand pediatric primary care-based literacy interventions, which have the potential to reach children long before they enter traditional educational avenues (44).

While healthcare providers agree on the importance of providing literacy education, many do not know where to begin, nor do they have the resources required to triage literacy and reading challenges. In a recent study of New Jersey general pediatricians, two-thirds of respondents felt unprepared to promote literacy due to their lack of knowledge regarding literacy resources. Only one-quarter of those surveyed participated in Reach Out and Read or an equivalent program, though half of those not participating expressed interest in including literacy assessment and intervention in their practice. Respondents stated that a lack of time, funding to support reading, and concerns regarding parents’ receptiveness prevent them from engaging in literacy promotion (45). Studies such as this one underscore the need to emphasize child-literacy interventions and resources in medical-school curricula and pediatrics residency programs, to ensure pediatricians feel more prepared and knowledgeable about literacy.

At a time when healthcare workers and educators alike are overwhelmed, technology may be a welcome solution to this challenge. With the increased prominence of telehealth following the onset of the COVID-19 pandemic, the development of online or virtual interventions has the potential for families to experience more frequent conversations around literacy with healthcare professionals. TipsByText, also known as Ready4K, delivers literacy interventions via text message to caregivers of children. Families received TipsByText interventions three times per week for seven months, including facts, encouragement, and reinforcements, beginning with the phrase “Doc says…” Researchers observed an equivalent three-month gain in literacy skills due to the TipsByText intervention (46). Such text-based programs have the potential to reach families more frequently, especially when combined with in-person programs implemented in pediatric primary-care settings.

## Looking forward

The documented relationship between childhood literacy and health outcomes points to a clear need for partnership among families, educators, and healthcare providers throughout childhood and adolescence. Existing programs like Reach Out and Read offer valuable insight into the impact of early literacy and underscore the important role pediatricians can play in delivering impactful early literacy guidance directly to families. These programs, along with new technological strategies like TipsByText provide the healthcare and education communities with proven, achievable models for a thriving partnership.

Moving forward, communities, schools, and organizations should work to engage stakeholders:

• *Healthcare Providers*: Advocating for and providing additional training and resources for pediatric medical professionals around child-literacy assessment, promotion, and intervention throughout childhood; integrating literacy as a measure of a child's health.
• *Educators*: Incorporating health centers in education settings when possible; providing professional development and age-appropriate content for early childhood and elementary educators to engage students in issues relating to health and literacy; and communicating with practitioners and families about health issues affecting students. Educators should continue to work with families and communities to provide a wide variety of books to foster positive reading habits in and out of school.
• *Families*: Educating parents and caregivers about the impact of early literacy and empowering families to create a literacy-rich home environment; providing families with the resources necessary to improve their mental and physical health.
• *Communities*: Increasing public messaging around the clear, lifelong link between literacy and health, ensuring that communities prioritize reading and books in discussions around public health issues.

By treating literacy as an urgent health issue, we can improve literacy rates and health outcomes in the United States, and ensure all children thrive—beginning today.

## Data Availability

The original contributions presented in the study are included in the article/Supplementary Material, further inquiries can be directed to the corresponding author.
