# Supplementary figures and images for: Reading for life-long health
Source: Front Pediatr. 2024 Jul 24;12:1401739. doi: 10.3389/fped.2024.1401739 (PMC11303134; doi:10.3389/fped.2024.1401739)

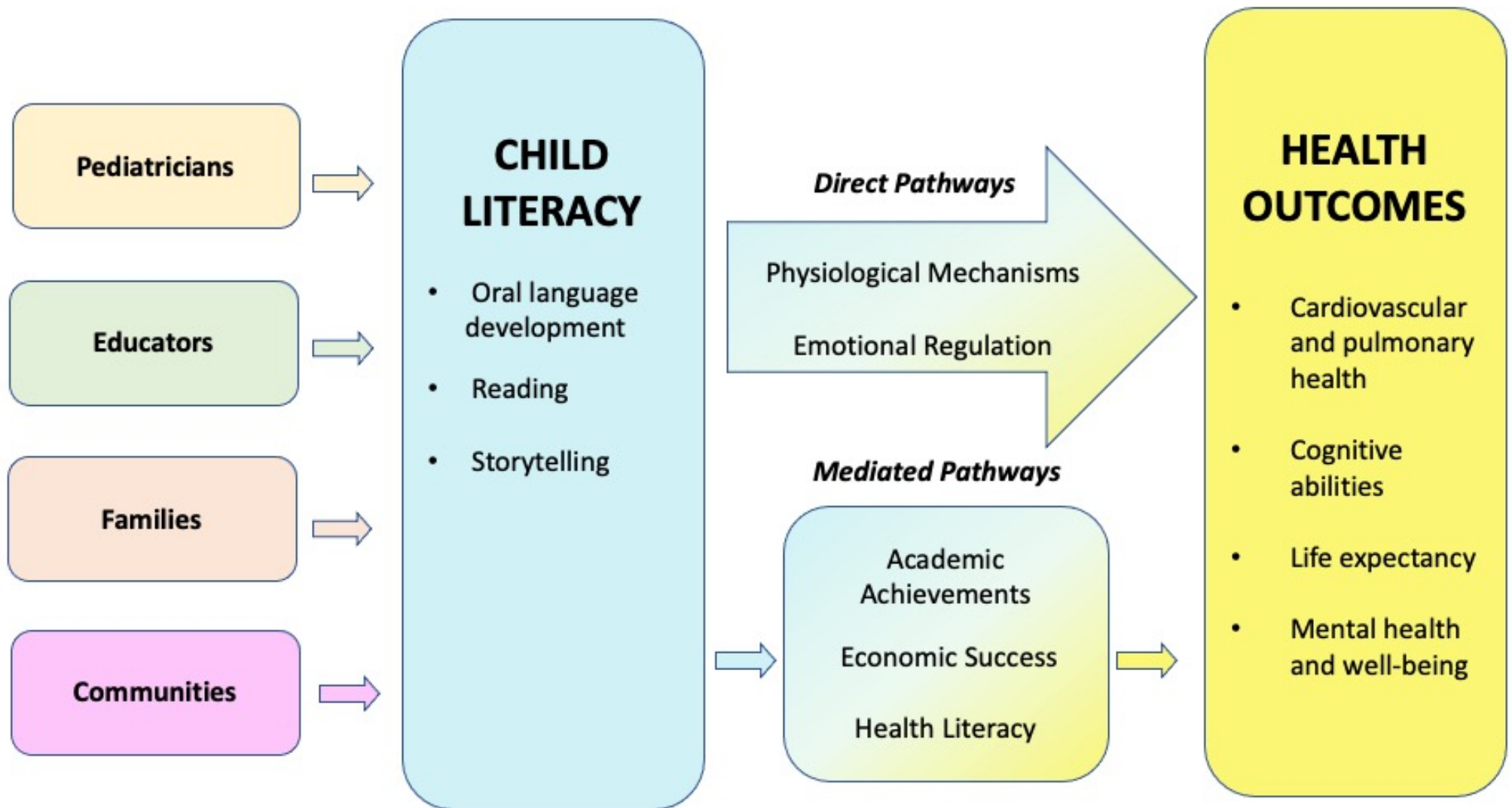

Supplement: Supplementary file 1 [file Image1.pdf]
